# A prospective study on the microbiological examination of secretions from the paranasal sinuses in horses in health and disease

**DOI:** 10.1186/s13028-018-0394-4

**Published:** 2018-07-05

**Authors:** Hauke Gergeleit, Jutta Verspohl, Judith Rohde, Karl Rohn, Bernhard Ohnesorge, Astrid Bienert-Zeit

**Affiliations:** 1 Clinic for Horses, University of Veterinary Medicine Hannover, Foundation, Bünteweg 9, 30559 Hannover, Germany; 2 Institute for Microbiology, University of Veterinary Medicine Hannover, Foundation, Bischofsholer Damm 15, 30173 Hannover, Germany; 3 Department of Biometry, Epidemiology and Information Processing, University of Veterinary Medicine Hannover, Foundation, Bünteweg 2, 30559 Hannover, Germany

**Keywords:** Anaerobes, Endoscopy, Horse, Microbiology, Sinusitis, *Streptococcus equi* ssp. *zooepidemicus*

## Abstract

**Background:**

Diagnostics in equine sinusitis can be challenging and often require a combination of different imaging tools to ascertain its underlying aetiology. The bacterial flora of healthy and diseased paranasal sinuses, respectively, has only sporadically been assessed in horses. The objectives of this study were to determine whether assessment of microbiological features of secretions from the paranasal sinuses displays a useful diagnostic tool in equine sinusitis to distinguish between different aetiologies. Secretion samples from 50 horses with sinusitis and from 10 healthy horses were taken transendoscopically from the drainage angle of the nasomaxillary aperture using a guidable Swing Tip catheter. Bacteria found in healthy and diseased equine sinuses were compared. Endoscopic samples in all healthy and 19 diseased horses were compared with samples taken directly from the affected sinus after trephination.

**Results:**

Eleven of the 14 horses with primary sinusitis revealed growth of *Streptococcus equi* ssp. *zooepidemicus*, with three samples yielding pure cultures. Anaerobes were found in 15 out of 26 samples from horses with dental sinusitis. Healthy sinuses revealed mainly α-haemolytic streptococci and coagulase-negative staphylococci or showed no growth. *Enterobacteriaceae* were found more frequently in secondary sinusitis. There were significant differences in the bacterial composition and diversity (*P* < 0.05) between primary sinusitis, dental sinusitis and healthy controls. The correlation between endoscopic and trephination samples was satisfying.

**Conclusions:**

Microbiological examination of secretions from horses with sinusitis collected transendoscopically can help to distinguish between primary and dental sinusitis. Therefore, it may display a feasible ancillary diagnostic tool, but does not replace a meticulous examination procedure including diagnostic imaging.

## Background

Sinusitis is a common and well described disorder in horses [1–3]. However, distinguishing between a primary sinusitis and those secondary to other disorders can be challenging. Although the diagnosis of sinusitis is usually easily made by assessment of clinical signs and endoscopic findings, an exact aetiological diagnosis can often only be achieved by a combination of several imaging diagnostic tools [3, 4]. In general practice, horses with unilateral nasal discharge are usually treated with antibiotics over varying periods of time and often without permanent remission of clinical symptoms [1]. This can make the healing process frustrating for the owner and the treating veterinarian.

The predominant organisms found in acute sinusitis in human medicine are *Haemophilus influenzae*, *Streptococcus pneumoniae* and *Moraxella catarrhalis*. A greater variation of bacteriological cultures exists in chronic sinusitis [5]. Maxillary sinusitis with an odontogenic origin in humans has been proven to be dominated by Gram-negative anaerobic bacteria [6]. The role of anaerobes has also been assessed for periodontitis, and endodontic and apical tooth infections in horses [7, 8], with reports of their involvement in equine dental sinusitis [9]. Nevertheless, the microbiological findings of secretions from the paranasal sinuses in horses have only been reported sporadically and have not yet been subject to wide case series or prospective studies [10].

The aims of this study were to assess whether the microbiological features of secretions from horses affected by sinusitis allow the drawing of conclusions regarding the underlying etiology and, therefore, whether this approach displays an ancillary diagnostic tool in equine sinusitis.

## Methods

### Patient group

The patient group (Table 1) consisted of 50 horses, including 39 Warmbloods, 5 ponies, 3 Icelandic horses, 1 English Thoroughbred, 1 Arabian Thoroughbred and 1 Coldblood (25 mares, 23 geldings and 2 stallions) with a mean ± SD age of 12.1 ± 6.3 years (range 1–27 years). They were presented to the Clinic for Horses of the University of Veterinary Medicine Hannover, Foundation, with a history of unilateral nasal discharge between October 2016 and September 2017. All horses were diagnosed with a paranasal sinusitis. Their history included the duration of clinical signs, previous medical and surgical treatments, and response to those treatments prior to referral. All horses underwent a detailed clinical examination and a specific clinical examination of the head.

**Table 1:** Distribution of the etiology of sinusitis

Aetiology	Number of cases
Dental sinusitis	26
Primary sinusitis	14
Paranasal sinus cyst	2
Progressive ethmoidal haematoma	2
Traumatic sinusitis	2
Conchal necrosis	1
Malignant neoplasia	3
Total	50

### Transnasal endoscopy and sampling

The endoscopic examination of the respiratory tract was performed using an Olympus video endoscope (PCF-H180AL).^1^ All horses were restrained in stocks and sedated using detomidine (Cepesedan,^2^ 0.01 mg/kg bwt i.v.) combined with butorphanol (Butorgesic,^3^ 0.01 mg/kg bwt i.v.).

**Fig. 1:**
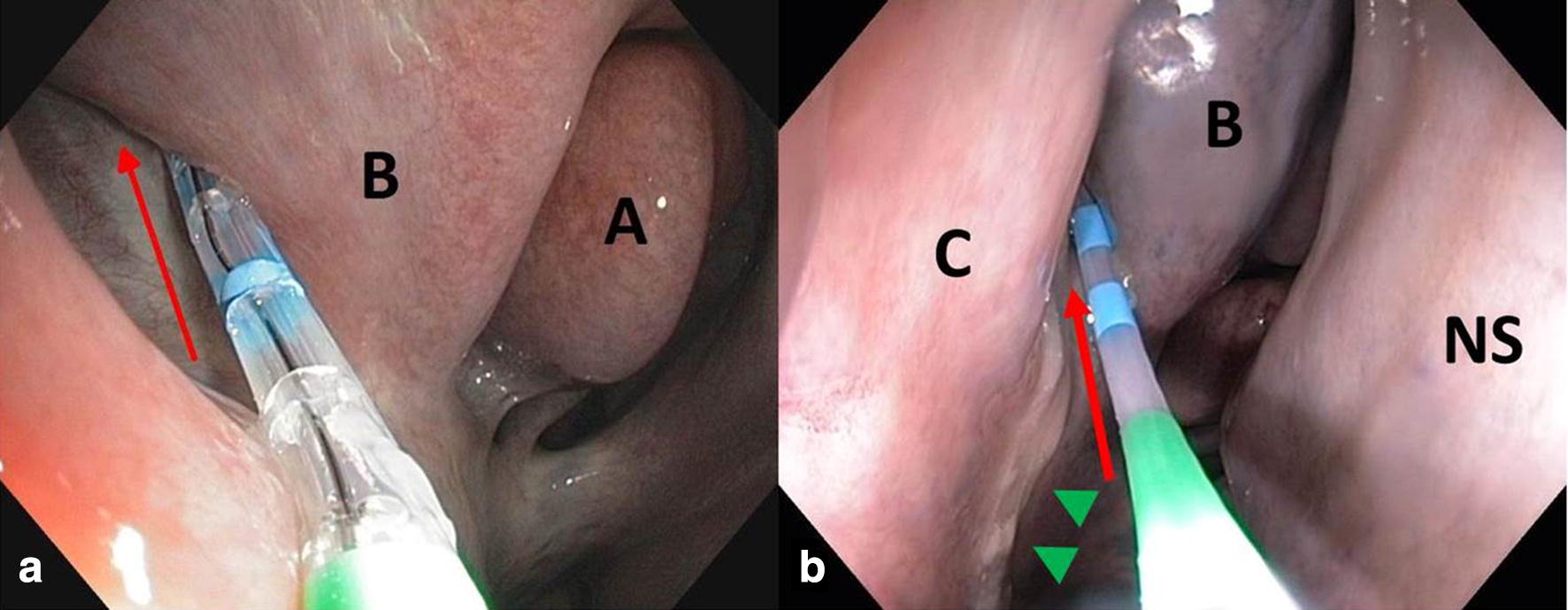
Endoscopic images of the right ‘drainage angle’ (red arrow) with the Swing Tip-catheter in place. The catheter is advanced along the drainage angle and secretions are aspirated. a) In a healthy control horse. b) In a horse with squamous cell carcinoma of the right sinus system with purulent, malodorous discharge (green arrowheads). A: middle nasal concha, B: dorsal nasal concha, C: ventral nasal concha, NS: nasal septum

Particular attention was paid to the nasal conchae, the nasal meati, with special regard to the caudal aspect of the middle nasal meatus (i.d. ‘drainage angle’), and the ethmoid region. Presence, quantity and nature of any secretion draining off the drainage angle were noted (Fig. 1). The endoscope was positioned in the middle nasal meatus rostral to the draining track. A flexible, wire-guided catheter (Olympus Swing Tip_TM_ PR-233Q^4^ [11]) was advanced through the working channel of the endoscope. Its distal tip was angulated using the handle attached until the catheter was positioned in the drainage angle and secretion was aspirated into the catheter. The catheter was removed before endoscopic examination was continued. The same method was used on both sides of the head in 35 horses to compare the findings between the usually unilaterally occurring symptoms in one nasal cavity and the non-affected side for an intra individual comparison. Airway endoscopy was completed to exclude other diseases.

Depending on history, clinical signs and findings during the endoscopic examination, further diagnostic tools including examination of the oral cavity, radiography and computed tomography of the head were combined to make an etiological diagnosis.

Nineteen horses underwent trephination or bone flap surgery of the diseased sinus for therapeutic reasons. In these cases a direct swab sample of the secretion or mass was taken for microbiological examination and compared to the sample collected transendoscopically to assess the method’s accuracy. The correlation between both samples was stated as good when they were dominated by the same microorganisms.

### Control group

The control group consisted of 10 horses, including 6 Warmbloods, 3 Trotters and 1 Icelandic horse (1 gelding and 9 mares) with a mean ± SD age of 16.6 ± 7.3 years (range 2–30 years) that were owned by the Clinic for Horses of the University of Veterinary Medicine Hannover, Foundation. The horses had no known history of sinonasal diseases and had not received antimicrobial drugs for at least 4 weeks prior to the study. They underwent a detailed clinical and radiographic examination of the head. The horses were put under general anesthesia for an unrelated terminal study. All procedures were approved by the Lower Saxony State Office for Consumer Protection and Food Safety (reference no. 33.19-42502-04-16/2212). Signs of sinonasal disorders were excluded during endoscopic examination before secretion samples were aspirated using the technique described previously. Afterwards, the ipsilateral frontal sinus was trephined under sterile conditions and a swab was inserted through the frontomaxillary opening to collect a mucosal smear from the caudal maxillary sinus for microbiological examination.

### Microbiological processing

The secretion specimens were applied to sterile cotton swabs that were placed into a semi-solid Amies transport medium^5^ and transferred to the Institute for Microbiology at the University of Veterinary Medicine Hannover, Foundation. The swabs were streaked on different solid media and incubated under aerobic, microaerophilic and anaerobic conditions. After streaking on solid media swabs were placed in enrichment broth which was incubated aerobically overnight and then plated on solid media. Further differentiation was achieved by following the Institute’s instructions for processing and examination. The degree of bacterial growth was assessed semi-quantitatively.

### Measures to decrease the risk of contamination

Precautions were taken to minimize the risk of contamination during transendoscopic sampling. In those horses that underwent transendoscopic sampling of both sides the sample from the clinically inconspicuous side was always taken first. We assumed lower bacterial burden for the control side and therefore minor risk of contamination on to the diseased side. The catheter with the sample was removed and the diseased side was sampled with a new, sterile catheter. Sterile gloves were used when handling the catheter. After each examination the endoscope and the catheters were thoroughly cleaned and disinfected according to the standards of the Clinic for Horses before they were used again. Each catheter was used approximately ten times before it was disposed.

### Statistical analysis

Statistical analysis was performed using the statistic software SAS, Version 9.4.^6^ Distinction of bacterial profiles between primary sinusitis, dental sinusitis and healthy controls was tested using Chi squared test. Fisher’s exact test was used to compare the prevalence of certain bacterial species and families between healthy and diseased sinuses. Wilcoxon–Mann–Whitney test was used to compare diversity between primary and secondary dental sinusitis as well as between healthy and diseased sinuses. *P*-values < 0.05 were considered statistically significant.

## Results

It was possible to place the catheter precisely in the drainage angle of the nasomaxillary aperture in all 60 horses. The amount and character of the secretions varied and secretions were more difficult to aspirate when they were of low quantity or high viscosity. The most common cause of sinusitis was dental disease (26 horses), followed by primary sinusitis (14 horses). Less common causes included neoplasia, sinus cysts, progressive ethmoidal haematoma (PEH), trauma and conchal necrosis (Table 1). Samples from healthy sinuses included the horses from the control group (n = 10) and from the contralateral, non-affected side in 35 horses from the diseased group. The bacterial profiles of horses with primary sinusitis, dental sinusitis and healthy controls were significantly distinct (*P* < 0.01).

### Primary sinusitis

The culture rate for horses with primary sinusitis was 100% with a median of two bacterial species per specimen (Min 1, Max 6, Range 5). *Streptococcus equi* ssp. *zooepidemicus* (*S. zooepidemicus*) was isolated transendoscopically in eleven horses, revealing pure growths in three cases and showing moderate to heavy growth in ten samples. *S. zooepidemicus* was isolated significantly more often in primary sinusitis than in dental sinusitis (*P* < 0.01) and healthy sinuses (*P* < 0.001) (Table 2). Moderate and high numbers of *S. zooepidemicus* were encountered significantly more often in primary than in dental sinusitis (*P* < 0.001). Eight horses had additional growth of Gram-negative bacteria, including two horses revealing *Enterobacteriaceae* and one horse with a heavy growth of *Pseudomonas aeruginosa*. One horse had a growth of mixed Gram-negative bacteria only. The antibiogram of the latter two horses showed the bacterial isolates to be resistant to the antibiotics used previously. Anaerobes were detected in only one horse with acute onset of primary sinusitis 3 days after undergoing a median laparotomy. There was a good correlation between the microbiological findings of endoscopic and trephination specimens (Table 4).

**Table 2:** Distribution of certain bacterial species among primary sinusitis, dental sinusitis and healthy sinuses

Primary sinusitis	n	*S. zooepidemicus*			Anaerobes			*Enterobacteriaceae*		
	14	11	78.6%	A*, B**	1	7.1%		2	14.3%	
Dental sinusitis	26	7	26.9%		15	57.7%	B**, C*	12	46.2%	B
Healthy sinuses	45	3	6.7%		0	0%		8	17.8%	

A: significant compared against dental sinusitis; B: significant compared against healthy sinuses; C: significant compared against primary sinusitis

* (*P* < 0.01); ** (*P* < 0.001)

### Dental sinusitis

The culture rate for horses with dental sinusitis was 100% for the samples collected transendoscopically. All samples revealed a median of three bacterial species (Min 1, Max 9, Range 8). Sinusitis with a dental origin showed a significantly higher diversity in the microbiological profile than those in primary sinusitis (*P* < 0.05) and healthy sinuses (*P* < 0.001). Fifteen samples showed mixed bacterial growth of facultatively and strictly anaerobic bacteria of which 13 showed growth of more than one anaerobic species. The frequent occurrence of obligate anaerobes is a significant difference to primary sinusitis (*P* < 0.01) and healthy controls (*P* < 0.001). Genera were dominated by *Fusobacterium* spp. and *Porpyhromonas* spp., and also included *Bacteroides* spp., *Prevotella* spp., Gram-negative and -positive obligatory anaerobes that could not be identified to the genus level, and *Peptostreptococcus* spp. (Table 3). The prevalence of anaerobic bacteria rises to 18 horses when horses in which anaerobes were detected from trephination samples are included.

**Table 3:** Number of strictly anaerobic isolates in dental sinusitis (n = 15)

*Bacteroides* spp.	1
*Bacteroides caccae*	1
*Bacteroides pyogenes*	2
*Fusobacterium* spp.	9
*Fusobacterium equinum*	1
*Fusobacterium necrophorum*	2
*Fusobacterium varium*	1
Gram-negative anaerobes	5
Gram-positive anaerobes	1
*Peptostreptococcus* spp.	2
*Prevotella* spp.	4
*Porphyromonas* spp.	6
Total	35

There was a higher prevalence of *Enterobacteriaceae* in horses with dental sinusitis (46.2%) than in those with primary sinusitis (14.3%), and a significantly higher prevalence than in healthy controls (*P* < 0.05), with *Escherichia coli* being encountered the most frequently. *S. zooepidemicus* was present in seven samples, with only two of them revealing moderate to high numbers.

### Other forms of sinusitis

Sinusitis that had an aetiology other than primary or odontogenic was rare in the current study. *S. zooepidemicus* was present in two cases of malignant neoplasia and in a paranasal sinus cyst, whereas a second case of a sinus cyst showed mixed facultatively anaerobic Gram-negative bacteria. One case of a squamous cell carcinoma of the right sinus system revealed growth of *Bacteroides fragilis*, *Porphyromonas* spp. and *Escherichia coli*. This horse had an extraction of 109 and 110 3 months prior to referral. Obligate anaerobic bacteria and *Enterobacteriaceae* were also present in a case of conchal necrosis. Sanguineous secretions in two cases of traumatic sinusitis due to fractures of frontal or maxillary bones revealed coagulase-negative staphylococci (CNS) in small numbers, whereas sanguineous nasal discharge in two horses in association with a PEH showed mixed facultatively anaerobic Gram-negative bacteria. The PEH itself did not yield any bacterial growth. These results cannot be analyzed statistically due to the small sample sizes.

### Healthy sinuses

The overall culture rate for healthy sinuses was 73.3% (33 out of 45 samples), with all positive cultures revealing a median of two bacterial species (Min 1, Max 5, Range 4). None of these samples revealed obligate anaerobes and only five showed low to moderate numbers of *Enterobacteriaceae*. The CNS and alpha-haemolytic streptococci (53.3% of positive cases) were the genera most frequently isolated. *S. zooepidemicus* was present in low numbers in three cases that were diagnosed with a primary sinusitis of the contralateral diseased side.

### Sinus surgery

Nineteen horses underwent therapeutic sinus surgery. Overall, there was a good correlation of the direct samples with the corresponding sample collected transendoscopically in 79% (Table 4). Bacteria that were estimated to play a dominant role in the endoscopic sample could also be verified in the direct swab sample in 15 out of 19 sample pairs. The endoscopic sample failed to reveal growth of anaerobic bacteria in comparison to the direct sample in three cases of dental sinusitis, whereas two other cases did not reveal anaerobic bacteria in either of the samples. There was good correlation of the endoscopic and the direct sample in all four horses with primary sinusitis. One horse with a paranasal sinus cyst was positive for *S. zooepidemicus* in both samples.

**Table 4:**
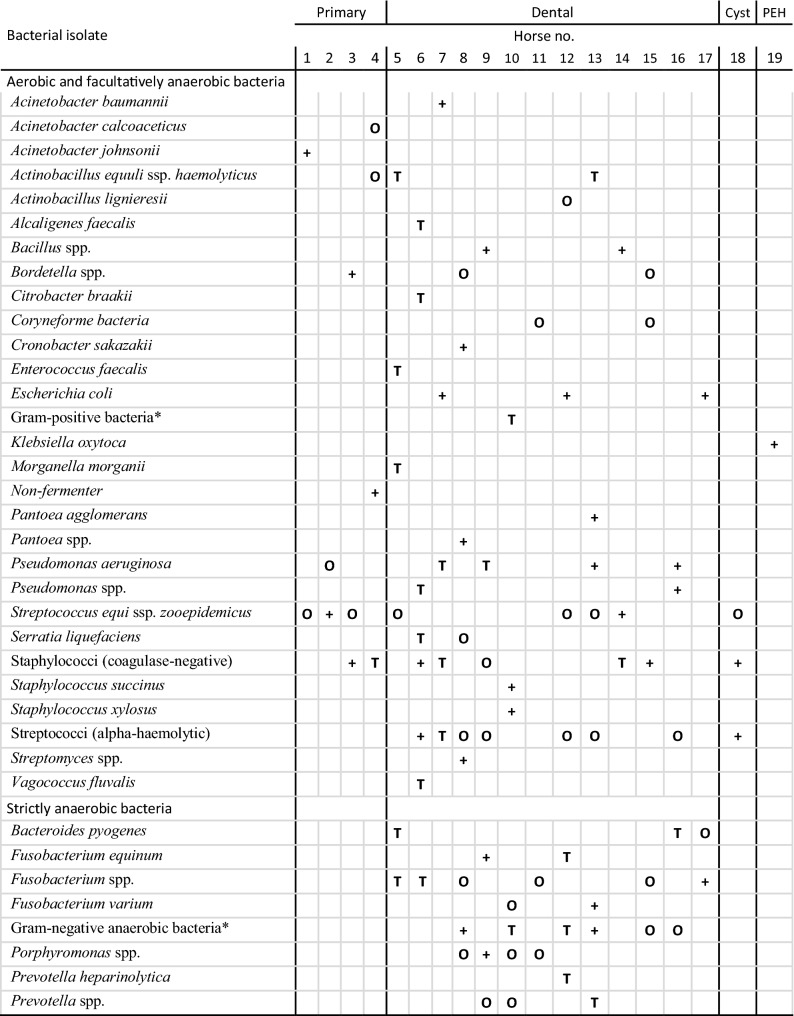
Bacterial isolates from transendoscopic samples and swabs taken directly after trephination

+, organism isolated from endoscopic sample; T, organism isolated from trephination sample; O, organism isolated from both: endoscopic and corresponding trephination sample; PEH, progressive ethmoidal haematoma

* Isolates could not be identified to the genus level

Samples that were taken from the caudal maxillary sinus after trephination in the control group did not reveal bacterial growth in six cases and low numbers of Gram-positive bacteria in four cases, whereas the corresponding transendoscopic samples showed mixed facultatively anaerobic Gram-negative and -positive bacteria in four horses and low numbers of Gram-positive bacteria and no bacterial growth in three horses each.

## Discussion

Diagnostics and successful treatment of sinusitis in horses can be challenging and frustrating to both the owner and the treating veterinarian in cases of prolonged and recurrent courses of this frequently described disease [3, 4]. Almost all cases included in this study had a history of chronic or recurrent nasal discharge of up to 5 years without permanent remission of symptoms with antibiotic treatment.

Symptoms of sinusitis were easily identified during clinical and endoscopic examination with varying severity of the disease. This is the first time that transendoscopic collection of secretions from the paranasal sinuses is reported. If discharge from the nasomaxillary aperture was present, the guidable Swing Tip catheter was easily positioned without causing mucosal bleedings. The use of the handle to control the bend of the tip before advancing the catheter along the drainage angle, facilitated sampling of secretions without gross contamination by touching the nasal mucosa or mucus present in the nasal cavity. In the absence of distinct discharge, it was possible to aspirate small amounts of serous secretions from the drainage angle.

Transendoscopic sampling for bacteriological culture holds the risk of contamination either through the endoscope or the catheter, if sterility of the utensils is not achieved, or through failure in handling. In this study, samples from the endoscope or the catheters to control for bacterial contamination were not taken, thus interference with the results cannot be fully excluded. Nevertheless the results indicate that our sampling and cleaning methods are extensively safe to avoid severe contaminations. The absence of bacteria or its detection in low numbers in most of the samples from the control group allows to draw conclusions that the risk of contamination was generally low. Moreover the findings between diseased and healthy sinuses differed significantly and some samples from horses with primary sinusitis even revealed pure cultures. However, the sample from the contralateral, non-affected sinus system in two horses revealed just as heavy growth of bacteria as the affected side, suggesting possible contamination between the two sides through the nasal cavity and the nasopharyngeal region. Undetected defects in the nasal septum display less often but are also possible reasons for these results [12]. Overall, the risk of interference with the results due to possible contamination can be regarded as low. The samples were taken following the same protocol and therefore had been exposed to an equal risk of contamination. Nevertheless, the bacterial findings between primary and dental sinusitis differed significantly in their composition and diversity, which remains the essential statement.

It was possible to obtain a direct sample during sinus surgery to assess the accuracy of the transendoscopic sampling method in 19 diseased horses. There was good correlation of the sample pairs in about 80% (Table 4), but complete consistency was not achieved in any of the sample pairs, probably due to the heterogeneous nature of secretions. Additionally, in most cases, 1 or 2 days passed between the endoscopic sampling and the trephination, during which antibiotic therapy had usually already been started. In three cases, the endoscopic samples failed to reveal anaerobes, whereas the corresponding trephination samples did. In one of these horses, characteristic secretion tracks along the drainage angle were absent, albeit large amounts of pus were present within the affected sinuses. Drainage was likely to be restricted due to inspissation of pus and narrowing of the drainage pathways. In the other two horses, purulent secretions with characteristic malodour could be collected. Since anaerobes have fastidious growth requirements, failure in handling, including prolonged storing, might have resulted in negative bacteriological culture.

*Streptococcus equi* ssp. *zooepidemicus* was the predominant organism associated with primary sinusitis in the current study, but was also isolated in association with some odontogenic and neoplastic conditions. These findings are similar to previous descriptions of this bacterial species in single cases of primary and dental sinusitis [4, 13, 14]. There was a distinctly smaller prevalence and it was less quantitative in secondary sinusitis overall. However, its presence in both primary and secondary sinusitis outlines the importance of diagnostic imaging in equine paranasal sinus diseases to exclude dental-related conditions and space-occupying processes. Microbiological evidence of *S. zooepidemicus* in two cases of malignant neoplasia and a paranasal sinus cyst in the current study indicate that its occurrence is not only related to primary inflammation of the sinus mucosa, but also to restricted sinonasal drainage, resulting in a reduction of mucociliary clearance [15]. *S. zooepidemicus* is often referred to as a mucosal commensal of the upper respiratory tract of healthy horses [16]. Nevertheless, none of the trephined healthy sinuses revealed growth of *S. zooepidemicus*, but it was detected in three endoscopic samples from healthy sinuses. These horses were diagnosed with a primary sinusitis of the contralateral sinus system with microbiological evidence of *S. zooepidemicus*, indicating possible contamination of the control side. *S. zooepidemicus* is a frequently isolated opportunistic pathogen that is often associated with severe pneumonia in young and middle-aged horses [16, 17]. The bacterium possesses numerous virulence factors, including adhesion capacities, and exerts an anti-phagocytic activity through the presence of hyaluronic acid and immunoglobulin-binding proteins [18]. The pathogenic potential of *S. zooepidemicus* is enhanced by the presence of pre-adapted variants of the bacterium that can adapt immediately to changes in its environment, such as alterations in the host’s immune status or transmission from a carrier horse to another susceptible individual [19].

Infections with *S. zooepidemicus* are not limited to the respiratory tract, but are also associated with septicaemia, placentitis, endometritis, peritonitis and arthritis, among others, in a wide range of species, including horses, livestock, dogs and camelids [20–22]. Although rarely described, transmissions from horses to humans are possible with cases of severe sepsis and meningitis, underlining the importance of recognizing this infection as a potential zoonosis [23]. The causative and highly contagious agent of strangles, *Streptococcus equi* ssp. *equi*, another closely related, but host-restricted β-haemolytic *streptococcus equi* subspecies with a much narrower tissue tropism [24], was not isolated in any of the samples of this study, although it had been described in equine sinusitis previously [4].

*Enterobacteriaceae* were isolated more frequently in horses with dental sinusitis than in healthy controls and in those with a primary sinusitis, although in the latter, these results did not reach statistical significance. Our findings outline parallels to human chronic sinusitis, where *Enterobacteriaceae* are considered as important infective agents with a secondary role [25].

Both aerobic and anaerobic bacteria were isolated from swab samples with a significant predominance of anaerobes in horses with dental sinusitis. The close relationship of the upper caudal cheek teeth to the maxillary sinuses makes the latter prone to becoming secondarily infected by dental diseases [2]. Obligatory anaerobic bacteria have been isolated previously from swab samples in horses affected by dental diseases [7, 8, 26] and have also been described in horses with dental sinusitis [9]. There is a good correlation between those findings and the results of this study. The microbiological profile of the horse with a conchal necrosis showed similarities to dental sinusitis, including anaerobic bacteria and *Enterobacteriaceae*. Notwithstanding, this horse had no known history of dental diseases and showed no radiological signs of a dental disease when presented, albeit malodorous unilateral nasal discharge was present indicating infection with strictly anaerobic bacteria. Historical, clinical and microbiological findings coincide with those of a case series about horses affected by conchal necrosis [27]. No anaerobes were found in the control group, either in the samples collected transendoscopically nor in the samples collected directly after trephination, suggesting that these organisms are not part of the physiological sinus flora in horses. However, they might be detectable using culture-independent broad platform molecular techniques. Results of microbiological findings in human medicine differ significantly in their diversity and composition when conventional cultivating methods are compared to molecular techniques [28].

The sole use of conventional bacterial culture methods displays a limitation of this study, since investigations in humans have shown that many bacteria cannot be cultured using conventional approaches [29]. Conventional culture methods and culture-independent analysis of the microbiological contents of middle nasal meatus specimens from humans with chronic rhinosinusitis showed broad consistency, but culture failed to detect potentially pathogenic bacteria [30]. Thus, it has been proven that the sequence-based approach provides a more precise phylogenetic resolution and detects greater biodiversity. Therefore, molecular genetic methods may also be beneficial for further investigations into the flora of equine paranasal sinuses.

The limiting factor with the biggest impact on this study’s outcome is probably the inhomogeneity of the patients group, with many horses being treated previously with various antimicrobial drugs over different periods of time. Prior antibiotic therapy is likely to cause incalculable alterations in the microbiological composition of the samples. Two cases of horses in this study with a primary sinusitis that were reluctant to multiple antibacterial drug administration, underline this theory. Both horses revealed Gram-negative bacteria that were resistant to the antibiotics that had been used previously. It would be desirable to repeat this study on horses without any previous antibacterial treatments to get more precise results.

The authors tried to identify specific pathogens in the present study that could serve as indicators for different parasinuidal diseases. However, the view of bacterial involvement in rhinosinusitis in human medicine has expanded beyond that of infection with individual pathogens to considerations of increased bacterial abundance, biofilm formation, intracellular bacteria and alterations in the microbial community (i.e. “microbiome”) [28]. Biofilm formation displays an important survival mechanism for microorganisms through attachment to surfaces and has been confirmed in human chronic sinusitis [31]. The extent to which bacteria in biofilms are cultivable has not been well studied in humans, and to the best of the authors’ knowledge, no attempts have been made to assess it in equine medicine. These bacterial survival mechanisms might not only add further variation to the detection of bacteria in this study and in microbiological examinations in equine practice in general, but may also play an essential role in recurring sinusitis and cases refractory to conservative therapy. It underlines the importance of surgical treatments and sinus irrigations to interrupt the vicious circle of impaired sinonasal drainage and progression of the disease.

Extensive longitudinal studies starting with healthy young horses and follow-up on their secretion and microbiota of the paranasal sinuses until development of sinusitis would be required to evaluate all these variable factors of host-microbial interactions.

## Conclusions

The results presented indicate that microbiological examination of secretions collected transendoscopically from horses with paranasal sinusitis can help to distinguish between primary sinusitis and those with a dental origin. Therefore, it may constitute a feasible ancillary diagnostic tool given that the sample is taken with the lowest possible risk of contamination. It is important to take the occurrence of diverse, polymicrobial cultures into consideration. However, microbiological examination does not replace a meticulous clinical examination of the head and the oral cavity and diagnostic imaging.

## Footnotes

1 Olympus Deutschland GmbH, Hamburg, Germany.

2 CP-Pharma Handelsgesellschaft mbH, Burgdorf, Germany.

3 CP-Pharma Handelsgesellschaft mbH, Burgdorf, Germany.

4 Olympus Deutschland GmbH, Hamburg, Germany.

5 Meus S.r.l, Piove di Sacco, Italy.

6 SAS Institute, Cary, NC, USA.
